# Fine mapping – 19th century style

**DOI:** 10.1186/1471-2156-6-S1-S63

**Published:** 2005-12-30

**Authors:** John Molitor, Keyan Zhao, Paul Marjoram

**Affiliations:** 1 Department of Preventive Medicine, University of Southern California, Los Angeles, CA 90089-9011, USA; 2 Molecular and Computational Biology, University of Southern California, University Park Campus, Los Angeles, CA 90089-1340, USA

## Abstract

**Background:**

There is great interest in the use of computationally intensive methods for fine mapping of marker data. In this paper we develop methods based upon ideas originally proposed 100 years ago in the context of spatial clustering.

**Methods:**

We use spatial clustering of haplotypes as a low-dimensional surrogate for the unobserved genealogy underlying a set of genotype data. In doing so we hope to avoid the computational complexity inherent in explicitly modelling details of the ancestry of the sample, while at the same time capturing the key correlations induced by that ancestry at a much lower computational cost.

**Results:**

We benchmark our methods using the simulated Genetic Analysis Workshop 14 data, using 100 replicates of 4 phenotypes to indicate the power of our method. When a functional mutation relating to a trait is actually present, we find evidence for that mutation in 97 out of 100 replicates, on average.

**Conclusion:**

Our results show that our method has the ability to accurately infer the location of functional mutations from unphased genotype data.

## Background

In this paper we present several applications of extensions of the method of Molitor et al. [1]. This method is itself based on ideas introduced by Georgy Voronoi at the turn of the last century [2]. Our application involves adaptations to Molitor et al. [1] that are designed to enable the analysis of data that are truly diploid. In this paper we present results of applying the methodology to the simulated data sets for Genetical Analysis Workshop 14 (GAW14). In doing so we hope to indicate the power of our method both in terms of determining that a functional mutation is present and then locating the mutation itself. We also begin to investigate the loss of signal caused by lack of phase information in genotype data. We refer readers to Molitor et al. [1] for all technical details of the original method. Due to space limitations, full details of the extension of this method to diploid data will appear in a subsequent methodologic paper. Our focus here is on application to the simulated GAW data.

The marker data resulting from a case-control sample, for example, are the result of the action of evolutionary forces such as recombination and mutation over the ancestral history of the sample. In principle this ancestral history can be described by a stochastic process known as the *coalescent *[3]. While the introduction of coalescent models has proven extremely powerful in many applications, these applications have primarily been in contexts in which recombination is absent and where the data can be assumed to have evolved without selective pressure. Neither assumption is likely to be valid for data appropriate for fine mapping studies. Furthermore, the complexity of such models in the presence of these complicating factors is enormous, and it is therefore entirely plausible that it is counter-productive to include them in analyses.

Therefore, there has recently been a move to consider approaches that attempt to approximate the key features of such models while avoiding most of the computational complexity. Some have attempted, with some success, to explicitly approximate aspects of the underlying coalescent process [4,5]. Others have used an approach that is more abstract in nature, in which the coalescent process is replaced by ideas borrowed from spatial statistics to produce a clustering of the data that, it is hoped, will capture some of the ancestral information in a way that is as simple as possible [1,6,7]. Analyses of the latter type are less complex in nature than those of the former type. Thus, while they might lose some power due to the use of a more abstract approximation to the underlying ancestry of the sample, they gain by imposing a smaller computational burden and are therefore likely to be able to analyze larger datasets. We believe both approaches are valid, but in this paper we focus on the more abstract methods.

We extend the methods of Molitor et al. [1] to contexts in which we are presented with diploid, rather than haploid data, and in which phase is unknown. We employ a Markov chain Monte Carlo (MCMC) algorithm in which haplotypes are reconstructed from the genotype data as part of the analysis, exploring the space of all likely haplotypes that are consistent with the genotype data as an explicit part of the fine mapping analysis. For a related approach that can analyze unphased genotype data see Lu et al. [8].

We believe that an integrated analysis is conceptually preferable to a two-stage approach in which haplotypes are first estimated and then the estimated haplotypes are used, as if they were known, as the basis of a separate fine mapping analysis. In an integrated analysis we use an MCMC algorithm to mix over the space of haplotypes consistent with the genotype data. If the identity of the haplotypes is of interest, one can estimate posterior distributions for them. However, in general, the primary interest will be in locating putative functional mutations within the genotypes. In this case, the uncertainty in the identification of haplotypes is explicitly included within the analysis, thus leading to more realistic estimates of certainty in the posterior distribution for the location of any functional mutations. See Clayton et al. [9] for a discussion of related issues. We employ the integrated method in this paper.

## Methods

We assume we have binary marker data at *J *loci for *I *diploid individuals, consisting of phenotypes *y*_*i*_; *i *= 1, ..., *I*, and genotypes *g*_*i *_= {*g*_*i*1_, *g*_*i*2_, ..., *g*_*iJ*_}, where *g*_*i *_= 0, 1, or 2, represents the number of copies of the less-frequent allele at locus *j *for individual *i*. We employ extensions to the model of [1] in which haplotypes are clustered according to ideas borrowed from spatial statistics. A full exposition of this methodology will appear in a future paper. In principle, our method adapts in a straightforward way to situations in which not all markers are SNPs, but for the sake of simplicity we have ignored non-binary markers in the analyses presented here.

At any given step of the MCMC algorithm, the set of genotype data will be resolved into a set of 2*I *haplotypes. Between iterations, we alter the way genotypes are resolved into haplotypes by taking a random subset of the loci for each genotype and reversing the way those loci are resolved for that genotype (so that alleles that were previously on the first of its two haplotypes will now be on the second haplotype and vice-versa). We let *h*_*ik*_; *k *= 1, 2 denote the two haplotypes into which genotype *g*_*i *_has been resolved. The set of all current haplotypes is then clustered according to a similarity measure based on shared-length identical by state (IBS). Each cluster, *c*, is determined by a cluster center *h*_*c*_, which is a haplotype, and *x*_*c*_, the location along the haplotype of a putative disease-associated mutation. Note that *x*_*c *_is free to take different values for different clusters at any given iteration. For a given set of cluster center haplotypes, we cluster each of our current sample haplotypes by calculating the shared lengths IBS at *x*_*c *_between each of the sample haplotypes and each of the haplotypes corresponding to a cluster center. Let *L*_*mn *_denote the shared length between sample haplotype *m *and center haplotype *n*. In a somewhat ad hoc attempt to avoid biases introduced by unequal marker spacing we define *L*_*mn *_as a ratio of observed and expected shared length at *x*_*c*_, where the expected shared length is calculated empirically from the observed data. As per the methods in Molitor et al. [1] we assign haplotypes to clusters in a deterministic manner, dependent upon shared length. Specifically, haplotype *m *is assigned to the cluster *n *for which the value of *L*_*mn *_is highest. In principle, one could use any other similarity metric, but clearly the power of the method will be adversely affected by using a metric that does not capture local haplotype similarity in an efficient way.

Each cluster has an associated parameter *γ*_*c *_that is used to define the expected trait value for haplotypes assigned to that cluster. We use this as the basis of a probabilistic assessment of the ability of the current clustering to explain the observed phenotypes. We let  denote the cluster to which *h*_*ij *_is assigned and write



where *α *represents an intercept term, *φ *is a variable that takes values between 0 and 1, *ε*_*i *_~ *N*(0, *σ*^2^) is an error term, and

*δ*(*γ*_1_, *γ*_2_, *φ*) = (1 - *φ*) min {*γ*_1_, *γ*_2_} + *φ *max {*γ*_1_, *γ*_2_}.     (2)

We mix over *φ *as part of the MCMC algorithm. *φ *can be thought of as indicating whether a recessive (*φ *= 0), dominant (*φ *= 1), or intermediate model is appropriate. When *φ *= 0, the fitted risk for an individual is determined by the minimum of the two haplotype risks and will therefore only take a high value if both haplotypes contain the functional mutation (and therefore have a high risk themselves). When *φ *= 1, individual risk will be high if either haplotype risk is high, which reflects a dominant scenario. When *φ *= 0:5, for example, we have an additive model. For the binary phenotypes we analyze in this paper we use a probit link in the above model.

The MCMC algorithm explores the parameter space corresponding to the model, including the number of clusters, cluster parameters and centers, assignment of genotypes to haplotypes, and imputed values for any missing marker information.

### Interpretation of output

An approach such as ours provides a full clustering of the data, as well as assignment of risks (i.e. *γ*_*c *_values) to haplotypes, and locations of putative functional mutations at each iteration. For each diploid individual we construct an empiric 95% confidence interval (CI) for the risk term *δ *associated with that individual in Equation (1). We did this by collating the *δ *values associated with that individual across all iterations (after the usual MCMC burn-in period) and constructing the smallest interval that contains the middle 95% of those values. We labeled a dataset as showing evidence for the presence of a functional mutation if there was any individual for which the 95% CI for *δ *does not overlap 0. For those datasets that show evidence for the presence of a functional mutation (according to the definition above) we then constructed a posterior distribution for the location of that functional mutation. We did this by recording the location *x*_*c *_associated with each cluster at each iteration of the algorithm. Each time a given *x*_*c *_value was observed, we added a weight of *w*_*c *_to the posterior distribution for location at *x*_*c*_, where *w*_*c *_is defined as the probability that a *N*(*γ*_*c*_, 1) random variable takes a value greater than 0. This weight is suggested by our use of a probit link function, where *P*(*y*_*i *_= 1) = Φ(*α *+ *γ*_*c*_), where Φ(·) is a standard normal cdf. Thus, locations corresponding to clusters with a high risk parameter were given high weight, whereas those corresponding to clusters with low risk were given low weight.

## Results and Discussion

**Figure 1:**
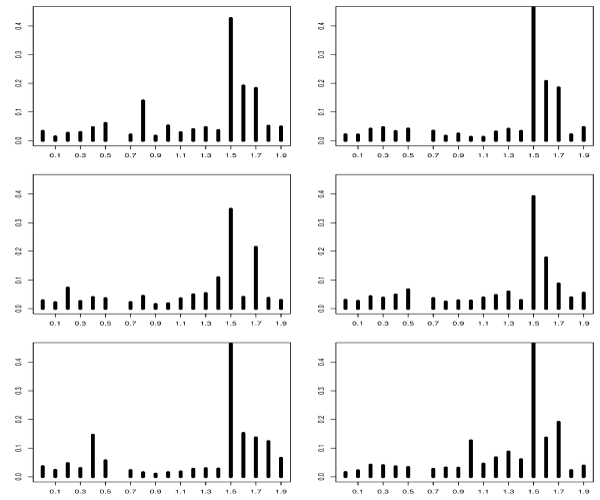
**Posterior distribution for functional mutation in first 6 replicates for phenotype e**. The figure shows the posterior distribution for the location of the functional mutation related to trait e in a phase-unknown analysis of each of the first 6 replicates of the packet 153 data.

We benchmark the methodologies presented in this paper via the GAW simulation study. We chose to analyze the GAW data knowing the answers. We present an analysis of traits e, f, g, and h for the Aipotu population using the packet 153 data. In an attempt to estimate the likely power of our method, we analyzed 100 replicates for each of these traits. We argue below that, due to the simulation method employed, the signal for the functional mutation for traits e, f, and h should be found in this packet, several SNPs in from the right-hand end. As an example of the output obtained from our method, in Figure 1 we give outputs from a phase-unknown analysis for the location of the functional mutation related to trait e in the first six replicates. Output for other replicates, and for analyses of traits f and h, are similar. Note that, in general, no individuals are found to be significant when analyzing trait g. This indicates that no evidence for a mutation related to trait g is indicated.

We note that our method makes no use of pedigree information when inferring phase. It treats the data as if it were a random sample from the population of interest. We chose to use all the data in each replicate, ignoring the pedigree information. As such, it is interesting to note that even when applied in an environment for which it is not explicitly designed, the algorithm appears to perform well and to be robust to departures from assumptions about the sampling scheme.

We summarize our results across all replicates as follows. For each phenotype we collected the analyses of the 100 replicates and recorded how often at least one genotype is found to be significant in the analysis. This was used as an indication of evidence that a functional mutation is present in the packet. In Table 1 we summarize how often a significant genotype was found for each of the phenotypes of interest across all 100 replicates. This gives us an indication of the power of our method. Our method found evidence of a functional mutation in almost all of the simulated datasets for which a functional mutation is present. Interestingly, for phenotype g, we found evidence of a significant genotype effect in 14 out of 100 replicates. This provides an estimate of the false-positive rate for our method. This number appears reasonable in light of the failure to allow for familial correlations. We constructed a 95% CI for the mean genotype risk for each individual, but there are many (heavily correlated) individuals, so our overall false-positive rate should be at least 5%.

Given that there is at least one significant individual, we inspected the posterior distribution for the location of the functional mutation for that replicate and record the marker that has highest posterior mass. We then collected this information for all 100 replicates for that phenotype. This gives us an indication of the accuracy of our method when a functional mutation is indicated. These summaries are shown in Table 2. We note that the location of the functional mutation is typically inferred with great accuracy. We argue below why the signal should be found a few loci in from the right-hand end of the region, around locus 16, rather than at the end itself. In the final column we report results for an analysis of trait e in which we pre-processed the data using the PEDPHASE program to infer haplotype phase information and then used a version of our program that assumes the inferred phases are true (and therefore does not explore other possible decompositions of genotypes into haplotypes). Such an analysis found evidence for a functional mutation in 97 of the 100 replicates, but we note that the location of the functional mutation appears to be inferred with less accuracy. Given the family-based nature of the simulation study, we can assume that PEDPHASE will infer haplotypes with a reasonable degree of accuracy. Therefore these results give some preliminary indication that the loss of signal when using our method on unphased genotype data is minimal. In contexts in which data represents a random sample from a population, or a case-control study, one might reasonably expect programs such as PEDPHASE to infer haplotypes with a much lower degree of accuracy, and that our more integrated analysis will therefore continue to have superior performance to approaches that directly analyze inferred haplotypes.

When there is no association between a trait and the packet under consideration, i.e., for trait g, we note that there appears to be a tendency to suggest a location toward the righthand end of the packet. When we repeatedly re-analyzed these datasets after randomly permuting the phenotypes we found no tendency for the analysis to suggest functional mutations in these (or any other particular) locations. This suggests that these (relatively few) spurious signals might be a consequence of the particular way in which this trait was simulated.

## Conclusion

In this paper we have demonstrated the potential of more 'heuristic' approaches to fine mapping. Such approaches aim to capture the correlations induced by the unobserved genealogy of a sample without incurring the computational burden that a full coalescence-based model would imply. For example, the analyses in this paper take approximately 3.5 hours each. In doing so we make it possible to analyze much larger datasets than are traditionally possible using coalescence-based methods. In particular, one might hope to use methods such as ours to perform a genome-wide scan. We will investigate this issue in future work.

We regard the results in this paper as highly encouraging and feel that heuristic methods such as ours offer the potential to be applicable to much larger datasets than more intensive, model-based models. The results also suggest that loss of power due to lack of phase information is minimal when using our method. With reference to the particular GAW datasets analyzed in this paper, we note that given the alphabetical sorting used to generate LD at locus D2, LD with the traits associated with this locus (i.e., e, f, and h) is expected to be highest somewhere towards the left-hand end of the sorted region (SNP locus B03T3056) and non-existent at the right-hand end of the region (i.e., SNP locus B03T3068, where the functional mutation was actually placed). Thus, a reasonable analysis method should expect to find a signal around B03T3056 rather than at B03T3068. This intuition is supported by the results in this and other papers.

Software used for these analyses are available from jmolitor@usc.edu.

## Abbreviations

CI: Confidence interval

GAW14: Genetic Analysis Workshop 14

IBS: Identical by state

MCMC: Markov chain Monte Carlo

## Authors' contributions

JM and PM were responsible for development of methodology. JM and KZ were responsible for code development and testing. KZ was responsible for the analysis of the GAW 14 data. The manuscript was written by PM.

## Figures and Tables

**Table 1:** Power study for Genetic Analysis Workshop data

Phenotype	e	f	g	h
Number of replicates with evidence of functional mutation	96	98	14	98

**Table 2:** Inferred functional locus across 100 replicates

Phenotype	e	f	g	h	e (inferred phase)
locus 16	81	78	7	75	70
locus 17	9	9	1	11	19
locus 18	3	7	4	6	2
other loci	3	4	2	6	6
